# Biliary tract instillation of a SMAC mimetic induces TRAIL-dependent acute sclerosing cholangitis-like injury in mice

**DOI:** 10.1038/cddis.2016.459

**Published:** 2017-01-05

**Authors:** Maria Eugenia Guicciardi, Anuradha Krishnan, Steven F Bronk, Petra Hirsova, Thomas S Griffith, Gregory J Gores

**Affiliations:** 1 Division of Gastroenterology and Hepatology, Mayo Clinic Center for Cell Signaling in Gastroenterology, Mayo Clinic, Rochester, MN, USA; 2 Department of Urology, University of Minnesota Twin Cities, Minneapolis, MN, USA

## Abstract

Primary sclerosing cholangitis (PSC) is a cholestatic liver disease of unknown etiopathogenesis characterized by fibrous cholangiopathy of large and small bile ducts. Systemic administration of a murine TNF-related apoptosis-inducing ligand (TRAIL) receptor agonist induces a sclerosing cholangitis injury in C57BL/6 mice, suggesting endogenous TRAIL may contribute to sclerosing cholangitis syndromes. Cellular inhibitor of apoptosis proteins (cIAP-1 and cIAP-2) are negative regulators of inflammation and TRAIL receptor signaling. We hypothesized that if endogenous TRAIL promotes sclerosing cholangitis, then cIAP depletion should also induce this biliary tract injury. Herein, we show that cIAP protein levels are reduced in the interlobular bile ducts of human PSC livers. Downregulation of cIAPs in normal human cholangiocytes *in vitro* by use of a SMAC mimetic (SM) induces moderate, ripoptosome-mediated apoptosis and RIP1-independent upregulation of proinflammatory cytokines and chemokines. Cytokine and chemokine expression was mediated by the non-canonical activation of NF-*κ*B. To investigate whether downregulation of cIAPs is linked to generation of a PSC-like phenotype, an SM was directly instilled into the mouse biliary tree. Twelve hours after biliary instillation, TUNEL-positive cholangiocytes were identified; 5 days later, PSC-like changes were observed in the SM-treated mice, including a fibrous cholangiopathy of the interlobular bile ducts, portal inflammation, significant elevation of serum markers of cholestasis and cholangiographic evidence of intrahepatic biliary tract injury. In contrast, TRAIL and TRAIL-receptor deficient mice showed no sign of cholangiopathy following SM intrabiliary injection. We conclude that *in vivo* antagonism of cIAPs in mouse biliary epithelial cells is sufficient to trigger cholangiocytes apoptosis and a proinflammatory response resulting in a fibrous cholangiopathy resembling human sclerosing cholangitis. Therefore, downregulation of cIAPs in PSC cholangiocytes may contribute to the development of the disease. Our results also indicate that inhibition of TRAIL signaling pathways may be beneficial in the treatment of PSC.

Primary sclerosing cholangitis (PSC) is a progressive, idiopathic cholangiopathy characterized by chronic inflammation of the biliary epithelium, resulting in obliterative fibrosis of intra- and extrahepatic bile ducts and chronic cholestasis.^1^ Persistent inflammation of the hepatic parenchyma and biliary tree eventually leads to cirrhosis and can predispose to the development of cholangiocarcinoma. Liver transplantation is currently the best curative option for patients with end-stage liver disease.^2^ The etiology of PSC is largely unknown and there are presently no effective pharmacologic therapies for this disease. Several animal models have been examined to gain insights into the pathogenesis of PSC and identify potential molecular targets for therapy, including the widely-utilized *Mdr2*^−/−^ mice. Although *Mdr2*^−/−^ mice develop a chronic PSC-like biliary phenotype,^3^ they do not mimic the human disease, as humans with PSC do not have defects in this gene nor loss of biliary phospholipid secretion into bile. Given the complex, multifactorial nature of the disease, none of the other animal models developed so far recapitulate all the characteristic features of PSC.^4^ Therefore, the development of new animal models of PSC remains critical for acquiring pathogenic insights and testing therapeutic strategies.

Previous studies demonstrated that systemic administration of a tumor necrosis factor-related apoptosis-inducing ligand (TRAIL) receptor agonistic antibody induces acute sclerosing cholangitis in C57BL/6 mice, with periductal fibrosis, biliary obstruction and bile duct loss resembling human PSC,^5^ implicating TRAIL receptor signaling in sclerosing cholangitis syndromes. Cellular inhibitor of apoptosis protein 1 and 2 (cIAP-1 and cIAP-2) negatively regulate TRAIL signaling,^6, 7^ suggesting that cIAP depletion in the biliary tracts may also promote sclerosing cholangitis syndromes in mice by potentiating TRAIL signaling. The major objective of this study was to examine this potential model of PSC and explore its pathogenesis.

cIAP-1 and cIAP-2 bind to multiple adaptor proteins and, through their E3 ubiquitin ligase activity, control ubiquitination-dependent signaling pathways, including NF-*κ*B.^8, 9^ NF-*κ*B is activated through two major signaling pathways, both of which are either positively or negatively regulated by cIAPs. These pathways are referred to as the canonical pathway, which is mediated primarily by the p65(RelA)/p50 dimer, and the non-canonical pathway, mediated by the RelB/p52 dimer.^10^ Triggering the canonical pathway results in the activation of the I*κ*B kinase (IKK) complex and leads to phosphorylation and proteasomal degradation of I*κ*B*α*.^11^ Loss of I*κ*B*α* allows the canonical NF-*κ*B dimer to translocate to the nucleus and promote transcription. Activation of the canonical pathway is a well-characterized response following death receptor engagement and negatively regulates pro-apoptotic signaling by facilitating expression of anti-apoptotic proteins. Thus, cIAP-1 and cIAP-2 cellular depletion, which prevents canonical NF-*κ*B activation, enhances TRAIL-mediated cell killing. In contrast, the non-canonical NF-*κ*B pathway is triggered by the accumulation and activation of NF-*κ*B-inducing kinase (NIK). In unstimulated cells, cIAP-1, cIAP-2 and TNF receptor associated factor 2 and 3 (TRAF2 and TRAF3) constitutively associate with NIK, resulting in rapid NIK proteasomal degradation due to ubiquitination by cIAP-1 and cIAP-2.^12, 13, 14, 15^ In the absence of cIAPs (or TRAF2 and/or TRAF3), NIK protein levels increase, leading to phosphorylation and activation of IKK*α* and IKK*α*-dependent NF-*κ*B2 (p100) partial proteasome processing to generate the active p52 subunit.^16^ The resulting RelB/p52 dimers then move to the nucleus and initiate gene transcription. Thus, depletion of cIAP-1 and cIAP-2 can promote TRAIL-mediated inflammation via activation of non-canonical NF-*κ*B pathway.^17^ Hence, cIAP depletion can result in either enhanced apoptosis and/or RelB/p52-dependent inflammation, and one or both of these pathways could cause sclerosing cholangitis.

The present study reports the observation that cIAP-1 and cIAP-2 are significantly downregulated in cholangiocytes of PSC patients. Depletion of cIAPs *in vitro* in normal human cholangiocyte cell lines is associated with NF-*κ*B-mediated upregulation of pro-inflammatory cytokines and chemokines. Loss of cIAPs in cholangiocytes *in vivo* following direct injection of a SMAC mimetic into the biliary tract of mice results in acute cholestatic liver injury with features of sclerosing cholangitis. Genetic disruption of TRAIL or TRAIL receptor completely prevents this injury, pointing to a key role for the TRAIL/TRAIL-receptor pathway in the development of sclerosing cholangitis. These results highlight the contribution of cIAPs and TRAIL/TRAIL-receptor signaling pathways in the pathogenesis of sclerosing cholangitis.

## Results

### cIAP-1 and cIAP-2 are downregulated in PSC

**Figure 1:**
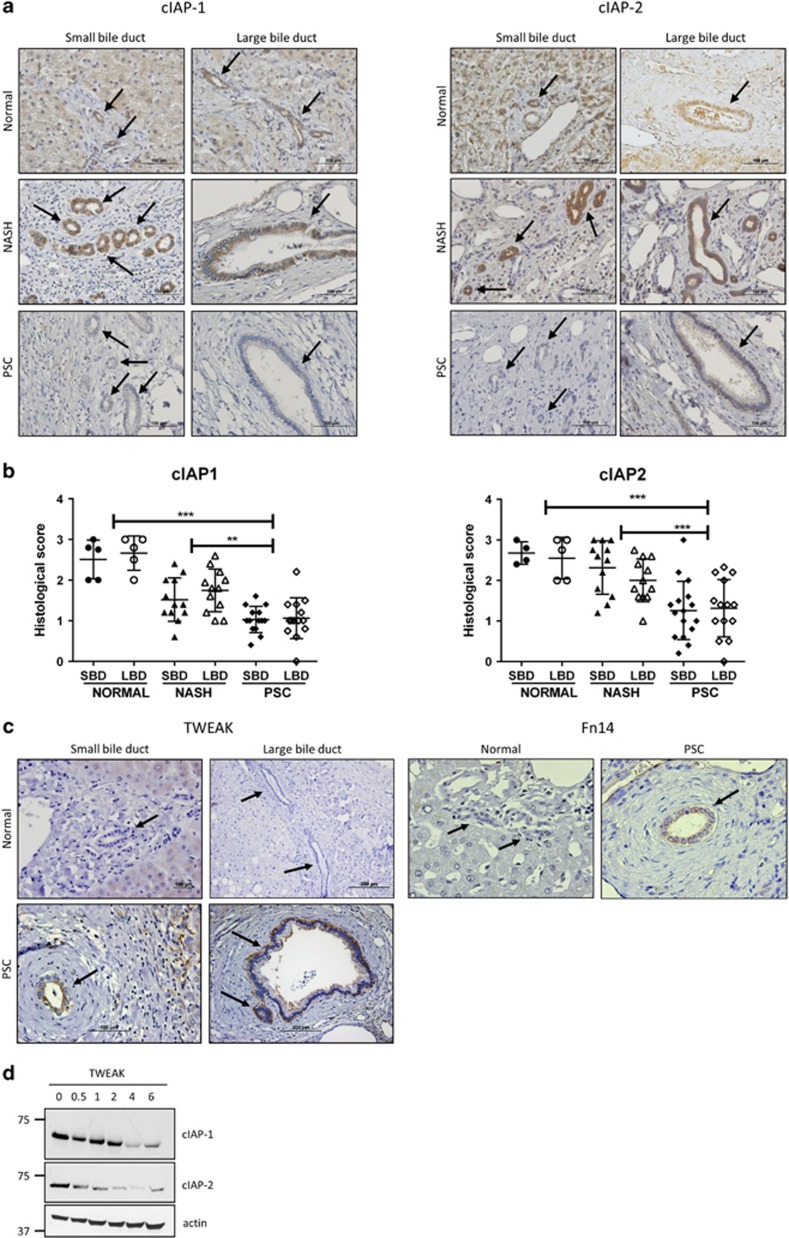
cIAP-1 and cIAP-2 are downregulated in cholangiocytes of PSC patients. (**a**) Representative images of liver sections stained for cIAP-1 (left panel) and cIAP-2 (right panel) in patients with normal, NASH and PSC (stage IV) liver histology. Photomicrographs of small bile ducts (SBD) and large bile ducts (LBD) taken at × 20 magnification. The arrows point to the bile ducts. (**b**) Histological scoring for cIAP-1 in normal (5, 31), NASH (12, 120) and PSC (16, 131) patients and for cIAP-2 in normal (5, 31), NASH (12, 118) and PSC (16, 123) patients. Numbers in parentheses indicate total number of patients and total number of small and large bile ducts evaluated, respectively. Grade 0=no protein expression; grade 3=high protein expression. **P<0.01, ***P<0.001. (**c**) Representative images of liver sections stained for TWEAK (left panel) and Fn14 (right panel) from patients with normal or PSC (stage IV) liver histology. Photomicrographs of small bile ducts and large bile ducts taken at × 40 and × 20 magnification, respectively. The arrows point to the bile ducts. (**d**) Immunoblot analysis showing expression of cIAP-1, cIAP-2 and actin (loading control) in H69 cells treated with human recombinant TWEAK (100 ng/ml) for the indicated times

Analysis of cIAP-1 and cIAP-2 protein expression in cirrhotic stage PSC patients revealed a significantly reduced cIAP-1 and cIAP-2 immunoreactivity in both small and large interlobular bile ducts compared to normal livers or disease control nonalcoholic steatohepatitis (NASH) livers (Figures 1a and b). Thus, downregulation of cIAPs in cholangiocytes appears to be a feature of, at least, advanced-stage PSC. TNF-like weak inducer of apoptosis (TWEAK) is a member of the TNF superfamily often induced in wounded tissues, where it promotes cytokine production and cell proliferation.^18^ Upon ligation to the TNF receptor superfamily member Fn14, TWEAK has been shown to induce lysosomal degradation of cIAP-1.^19^ To investigate whether loss of cIAPs may be triggered by TWEAK, we performed immunohistochemistry for TWEAK and Fn14 on normal and PSC liver sections. TWEAK immunoreactivity was detected in both small and large bile ducts in 78% (21/27) of PSC liver sections, but not in normal livers (0/5; Figure 1c). Similarly, Fn14 was expressed in PSC cholangiocytes, but not in normal cholangiocytes (Figure 1c). In addition, the incubation of H69 human cholangiocytes with recombinant TWEAK resulted in cIAP-1 and cIAP-2 degradation within 4 h (Figure 1d). Therefore, loss of cIAPs in PSC cholangiocytes may result from their degradation through activation of a TWEAK/Fn14 signaling pathway. Loss of cIAPs may promote caspase-mediated cell death and/or NF-*κ*B-driven cytokine and chemokine secretion by distinct signaling pathways relevant to sclerosing cholangitis. Therefore, we next explored these signaling pathways *in vitro* in cholangiocyte cell lines.

### Normal cholangiocytes are moderately sensitive to SM-induced, ripoptosome-mediated apoptosis

**Figure 2:**
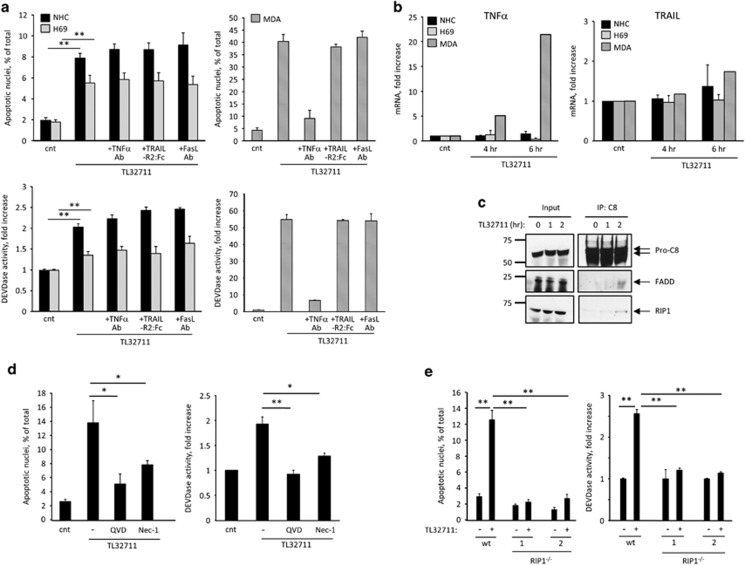
Normal human cholangiocytes are moderately sensitive to SM-induced, ripoptosome-mediated apoptosis. (**a**) Apoptosis assessed morphologically after DAPI staining (top panels) and by caspase 3/7 activation (bottom panels) in human cholangiocyte cell lines H69 and NHC, and the human breast cancer cell line MDA-MB-231, incubated for 24 h with or without (cnt) the SMAC mimetic TL32711 (1 *μ*M), in the presence or absence of neutralizing antibodies against TNF*α* (1 *μ*g/ml) or FasL (1 *μ*g/ml), or recombinant TRAIL-R2:Fc (1 *μ*g/ml). (**b**) *TNFα* and *TRAIL* gene expression analyzed by qPCR in H69, NHC and MDA-MB-231 cells incubated in the presence of TL32711 for the indicated times. Expression normalized to GAPDH RNA. Data are expressed as fold increase over control. Mean±s.e. are depicted from three independent experiments in H69 and NHC (**c**) H69 cells were treated with TL32711 and subjected to caspase 8 immunoprecipitation at the indicated time points. Affinity-purified proteins and total cell lysates (input) were analyzed by immunoblot for caspase 8, FADD and RIP1. (**d**) NHC cells were incubated with or without the TL32711 for 24 h, in the presence or absence of the pan-caspase inhibitor Q-VD-OPh (QVD, 10 *μ*M) or the RIP1 kinase inhibitor necrostatin 1 (nec-1, 20 *μ*M). Apoptosis was assessed morphologically after DAPI staining (left panel) and by caspase 3/7 activation (right panel). (**e**) H69 and *RIP1*^−/−^ H69 (clones 1 and 2) were incubated for 24 h in the presence or absence of TL32711. Apoptosis was assessed by DAPI staining (left panel) and by caspase 3/7 activation (right panel). In (**a**, **d** and **e**), mean±S.E. are depicted from three independent experiments performed in triplicate. *P<0.05, **P<0.005

First, the human cholangiocyte cell lines H69 and NHC were incubated with the bivalent SMAC mimetic (SM) compounds TL32711 (birinapant, 1 *μ*M) or BV6 (5 *μ*M). Although both compounds induce rapid degradation of cIAP-1 and cIAP-2,^12, 13, 20^ TL32711 is generally less efficient in inducing cell death due to its lower affinity for X-linked IAP (XIAP).^21^ In both the cell lines, the SM induced modest cell death, with less than 10% of the cells undergoing apoptosis after 24 h (Figure 2a and Supplementary Figure S2A). As SM-induced apoptosis can depend on autocrine TNF*α* signaling,^12, 13^ the cells were also treated with the SM in the presence of an anti-TNFα neutralizing antibody. No significant differences in cell death were identified in the presence or absence of the neutralizing antibody, nor was *TNFα* gene expression altered following the SM treatment, suggesting that SM-induced apoptosis in normal cholangiocytes is not mediated by autocrine production of TNFα (Figures 2a and b,Supplementary Figures S2A and B). Conversely, the SM-sensitive cell line MDA-MB-231 upregulated *TNFα* transcription and underwent substantial apoptosis in response to the SM, as previously reported^13^ (Figures 2a and b,Supplementary Figures S2A and B). These results suggest that SM may induce cholangiocyte apoptosis alternatively through formation of the ripoptosome.^22^ To examine this possibility, we first ruled out the involvement of the death ligands TRAIL and FasL/CD95L by incubating the cholangiocyte cell lines with either TL32711 or BV6 in the presence or absence of recombinant TRAIL-R2:Fc or a neutralizing antibody against FasL/CD95L. Similarly to TNF*α*, inhibition of TRAIL or FasL/CD95L failed to prevent apoptosis induced by the SM (Figure 2a and Supplementary Figure S2A). Accordingly, *TRAIL* expression was not induced by the SM treatment, whereas *FasL/CD95L* expression was undetectable in both treated and untreated cholangiocyte cell lines (Figure 2b and Supplementary Figure S2B). We next immunoprecipitated caspase 8 from untreated and SM-treated cells and analyzed the copurified proteins by immunoblot. Both the ripoptosome's core components Fas-associated protein with death domain (FADD) and receptor-interacting protein 1 (RIP1) were identified in association with caspase 8 after 2 h of SM treatment (Figure 2c). The inhibitor of RIP1 kinase activity necrostatin-1 significantly reduced SM-induced apoptosis (Figure 2d), consistent with the observation that ripoptosome-mediated cell death requires RIP1 kinase activity, as previously reported.^22^ In addition, we generated CRISPR/Cas9-targeted *RIP1*^−/−^ H69 cells (Figure 4b) and evaluated the effect of loss of RIP1 on SM-induced apoptosis. The results confirmed the essential role of RIP1, as RIP1 deficient cells were completely protected against SM-induced apoptosis (Figure 2e). Taken together, these results indicate that loss of cIAPs in normal cholangiocytes triggers a modest apoptotic response mediated by assembly of the ripoptosome.

### SM induces upregulation of pro-inflammatory cytokines and chemokines in human cholangiocytes independent of RIP1

**Figure 3:**
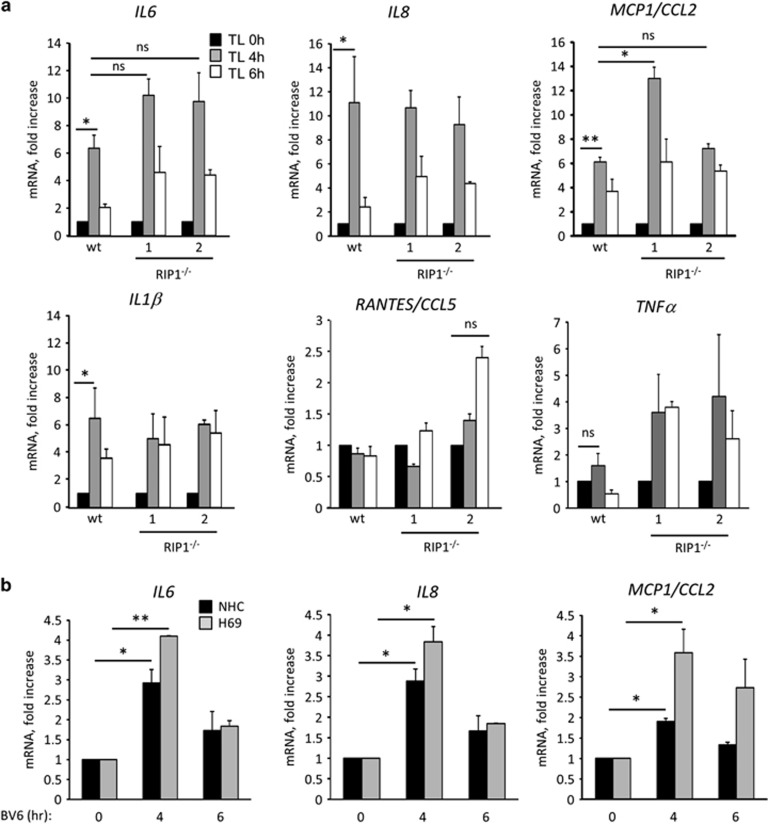
SM induces upregulation of pro-inflammatory cytokines independent of RIP1. (**a**) *IL6*, *IL8*, *MCP1/CCL2*, *IL1β*, *RANTES/CCL5* and *TNFα* gene expression analyzed by qPCR in H69 and *RIP1*^−/−^ H69 (clones 1 and 2) incubated in the presence of TL32711 for the indicated times. Expression normalized to 18S rRNA. (**b**) *IL6*, *IL8* and *MCP1/CCL2* gene expression analyzed by qPCR in H69 and NHC incubated in the presence of BV6 (5 *μ*M) at the indicated times. Expression normalized to GAPDH RNA. Data are expressed as fold increase over control. Mean±S.E. are depicted from three independent experiments. *P<0.05, **P<0.005; NS, not significant

SM treatment can trigger the production of chemokines, in particular RANTES/CCL5 and IL-8, without causing cell death.^23^ To determine whether SM treatment of normal cholangiocytes lead to the induction of cytokines and chemokines, H69 and NHC were incubated with TL32711 or BV6 and gene expression of several cytokines and chemokines were analyzed by qPCR. The results indicated that transcription of *IL6*, *IL8*, *MCP1/CCL2* and *IL1β* genes was significantly increased following SM treatment, peaking at 4 h and decreasing thereafter, whereas *RANTES/CCL5* and *TNFα* were unchanged (Figures 3a and b). In contrast to the apoptosis resistance displayed by *RIP1*^−/−^ cells, loss of RIP1 did not impair the SM-induced cytokine and chemokine production (Figure 3a), suggesting that at least two distinct signaling pathways are activated in response to the SM in cholangiocytes.

### SM-induced upregulation of pro-inflammatory cytokines and chemokines is mediated by the non-canonical NF-*κ*B pathway

**Figure 4:**
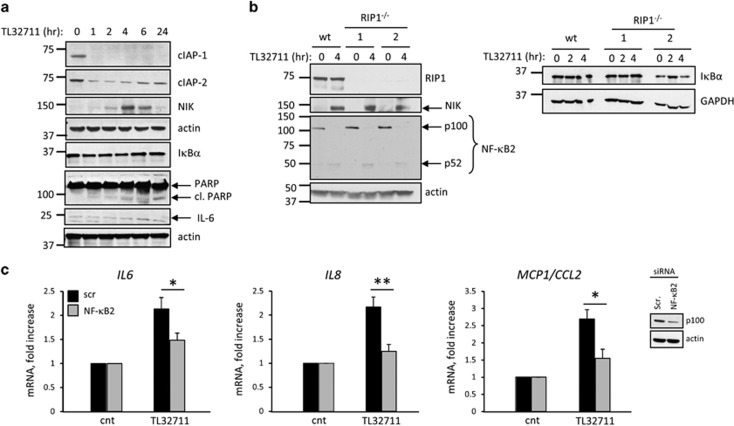
SM-induced upregulation of pro-inflammatory cytokines is mediated by activation of the non-canonical NF-*κ*B pathway. (**a**) Immunoblot analysis showing expression of cIAP-1, cIAP-2, NIK, I*κ*B*α*, PARP, IL-6 and actin (loading control) in H69 cells treated with TL32711 for the indicated times. (**b**) Immunoblot analysis showing expression of RIP1, NIK, NF-*κ*B2, actin (loading control), I*κ*B*α* and GAPDH (loading control) in H69 and *RIP1*^−/−^ H69 cells treated with TL32711 for the indicated times. (**c**) *IL6*, *IL8* and *MCP1/CCL2* gene expression analyzed by qPCR in NHC cell line transiently transfected with siRNA against NF-*κ*B2 (p100) or scrambled siRNA for 48 h and incubated in the presence or absence (cnt) of TL32711 for 4 h. Efficiency of the knockdown was evaluated by immunoblot (insert). Data are expressed as fold increase over control. Mean±S.E. are depicted from six independent experiments. *P<0.05, **P<0.005

*IL6*, *IL8*, *MCP1/CCL2* and *IL1β* are all known targets of NF-*κ*B. To determine whether SM-induced upregulation of these pro-inflammatory cytokines and chemokines was mediated by activation of NF-*κ*B, we initially performed immunoblot analysis on cell lysates from H69 cells treated with the SM over a 24 h time course. Activation of the non-canonical NF-*κ*B pathway was confirmed by the stabilization and accumulation of NIK and processing of NF-*κ*B2 p100 into p52 (Figures 4a and b). As previously reported, NIK levels rose rapidly upon inhibition of cIAPs and decreased again as cIAP2 levels were upregulated in response to non-canonical NF-*κ*B activation.^12, 13, 24^ Conversely, the canonical pathway of NF-*κ*B was not activated, as no degradation of the inhibitory subunit I*κ*B*α* was observed (Figures 4a and b). More importantly, siRNA-mediated knockdown of NF-*κ*B2 significantly attenuated cytokine and chemokine upregulation (Figure 4c), demonstrating that their transcriptional activity is largely regulated by the non-canonical NF-*κ*B pathway.

### Intrabiliary instillation of a SM in mice causes a PSC-like phenotype

**Figure 5:**
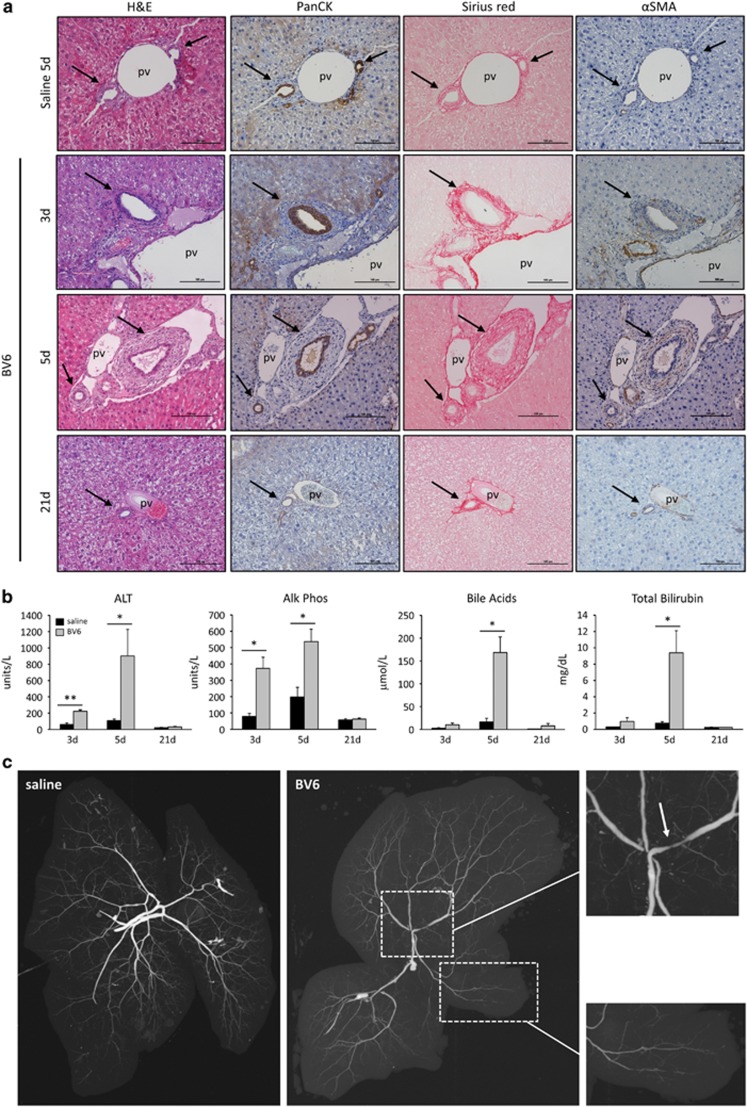
SM intrabiliary instillation in mice results in a transient PSC-like liver injury. (**a**) Representative images of liver sections from mice injected with a single dose of BV6 in the biliary tree and killed after 3, 5 or 21 days; and mice injected with saline and killed after 5 days. Hematoxylin–eosin (H&E)-staining, cholangiocyte staining by panCK IHC, collagen staining by picrosirius red-stain and *α*-smooth muscle actin (*α*SMA) IHC are depicted. Photomicrographs taken at × 20 magnification. The arrows point to the bile ducts; pv, portal vein. (**b**) Serum alanine aminotransferase (ALT), alkaline phosphatase (Alk Phos), total bile acids and total bilirubin measured in mice killed 3, 5 or 21 days after a single dose intrabiliary injection of BV6. Day 3: saline (*n*=4), BV6 (*n*=6); day 5: saline (*n*=8), BV6 (*n*=8); day 21: saline (*n*=6), BV6 (*n*=5). *P<0.05, **P<0.005. (**c**) Representative cholangiographic images of mouse livers 5 days after intrabiliary injection of saline or BV6. Enlargements of the indicated areas are shown next to the original picture. The arrow in the top magnification panel shows a stricture in an intrahepatic duct and adjacent dilatation. The bottom magnification panel shows damage/loss of small bile ducts. The gall bladder was resected to facilitate optimal visualization of the biliary tree during the cholangiograms

**Figure 6:**
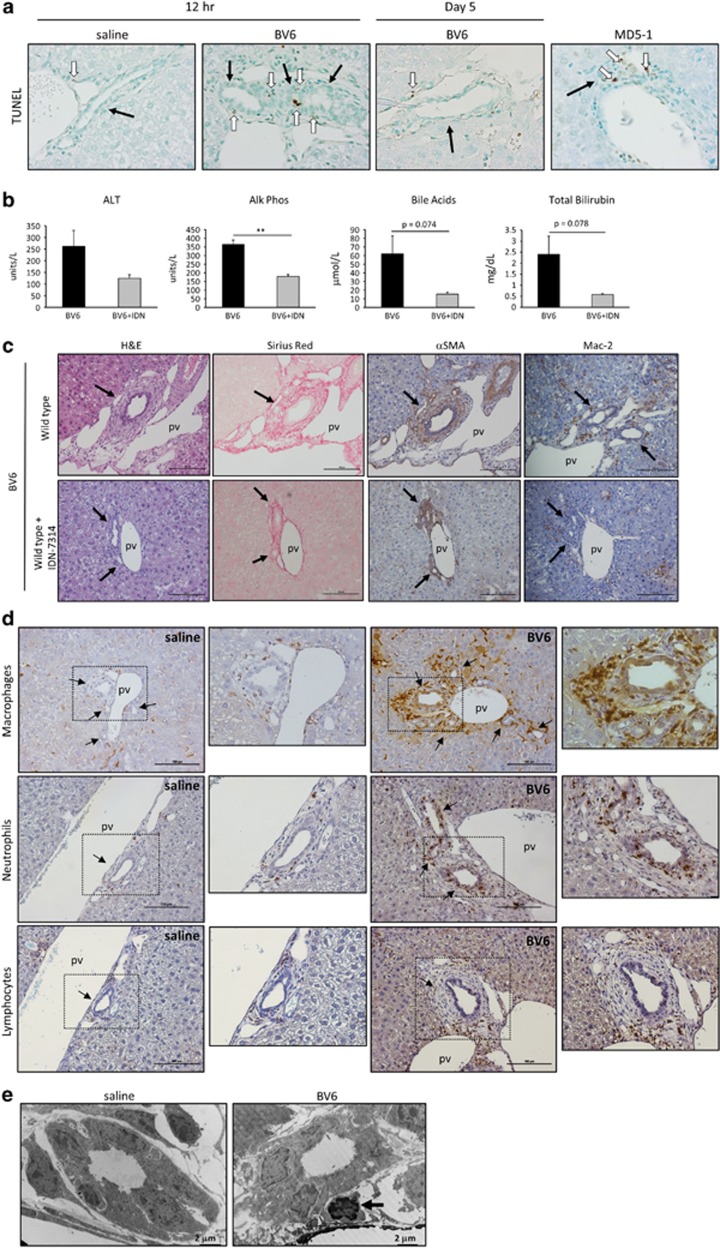
SM intrabiliary instillation in mice causes early cholangiocyte apoptosis and triggers periductal inflammation. (**a**) Representative images of TUNEL staining on fixed mouse liver specimens 12 h after intrabiliary injection of saline or BV6, 5 days after intrabiliary injection of BV6, or 4 days after systemic injection of MD5-1. The latter mice received two consecutive injections of MD5-1 (300 *μ*g/mouse) on day 1 and day 4 and were killed at day 8. This experimental paradigm induces cholangiocyte apoptosis and was used as positive control.^5^ The black arrows point to the bile ducts; the white arrows point to TUNEL-positive/apoptotic cholangiocytes. Photomicrographs taken at × 40 magnification. (**b**) Serum ALT, alkaline phosphatase, total bile acids and total bilirubin measured in mice killed 5 days after intrabiliary instillation of BV6 or BV6+IDN-7314. BV6 (*n*=7); BV6+IDN-7314 (*n*=5). **P<0.005. (**c**) Representative images of H&E staining, collagen staining by picrosirius red-stain and immunohistochemical staining for *α*-smooth muscle actin and Mac-2/galectin3 (marker of phagocytically active macrophages) on liver sections of mice 5 days after intrabiliary instillation of BV6 or BV6+IDN-7314. The arrows point to the bile ducts. Photomicrographs taken at × 20 magnification; pv, portal vein. (**d**) Representative images of immunohistochemical staining for Mac-2/Galectin 3 (macrophages, top panel), mouse neutrophil differentiation antigen (neutrophils, middle panel) and CD3 (lymphocytes, bottom panel) on fixed mouse liver specimens 5 days after intrabiliary instillation of saline or BV6. Photomicrographs taken at × 20 magnification. Enlargements of the indicated areas are shown next to the original pictures; pv, portal vein. (**e**) Representative transmission electron microscopy (TEM) images of mouse liver specimens 5 days after intrabiliary injection of saline or BV6. The arrow points to a macrophage adjacent to the bile duct

To establish whether downregulation of cIAPs in PSC cholangiocytes actively contributes to the pathogenesis of sclerosing cholangitis, we directly injected BV6 into the biliary tree of C57BL/6 mice. The effect of the drug on the biliary system was monitored over a period of 3 weeks during which the mice were killed at different time intervals. The first signs of biliary injury were observed at day 3 and appeared to peak at day 5. Complete regression of the injury with normalization of serum markers and histology was achieved by day 21 (Figures 5a and b). On the basis of these observations, the mice were killed at day 5 for the rest of the study to examine the full extent of the injury. At this time point, the livers displayed histological features consistent with a fibrous cholangiopathy of the interlobular bile ducts, as indicated by the classic concentric ductal fibrosis ('onion skinning') of the bile ducts within portal tract areas, and positive staining for activated myofibroblast and collagen (Figure 5a). Inflammatory cell infiltration was evident around the bile ducts (Figure 5a). BV6-injected mice also displayed significant elevation of serum alkaline phosphatase, total bile acids, bilirubin and alanine aminotransferase (ALT; Figure 5b), consistent with a cholestatic liver injury. Cholangiographic evidence of intrahepatic biliary strictures and dilatations, together with damage and loss of the small bile ducts, were also observed (Figure 5c). Analysis of bile ducts by electron microscopy and TUNEL staining showed no evidence of cholangiocyte apoptosis in BV6-injected livers after 5 days (Figures 6a and e). However, TUNEL-positive cholangiocytes were clearly identified in the livers 12 h after the intrabiliary instillation of BV6 (Figure 6a), suggesting that the SM elicits an early apoptotic response in the exposed cholangiocytes. Early cholangiocyte apoptosis, and possibly apoptosis of other neighboring cells, likely contributes to the subsequent biliary tract inflammation and injury, as simultaneous administration of the pan-caspase inhibitor IDN-7314 partially attenuates the hepatobiliary injury 5 days after treatment with BV6 (Figures 6b and c). Surprisingly, co-injection of Nec-1 s (10 *μ*M) with BV6 did not significantly prevent the injury, suggesting that RIP1 kinase activity is not required for SM-induced cholangiocyte apoptosis and/or hepatobiliary injury in this *in vivo* model (data not shown). These data also suggest necroptosis does not contribute to the injury observed in this model, an interpretation consistent with protection by the caspase inhibitor. Apoptotic cholangiocytes are probably engulfed by infiltrating macrophages and are therefore undetectable by day 5. Indeed, infiltration and accumulation of macrophages around the bile ducts was easily detectable in BV6-injected livers, but was decreased in mice simultaneously treated with the caspase inhibitor (Figures 6c–e). In addition to macrophages, other inflammatory cells (neutrophils, B and T lymphocytes) also accumulated around the bile ducts in BV6-injected livers (Figure 6d). Thus, SM injection in the biliary tree in mice triggers both an apoptotic and an inflammatory response.

### Activation of the TRAIL/TRAIL-R signaling pathway is required for cholestatic liver injury following biliary instillation of an SM

**Figure 7:**
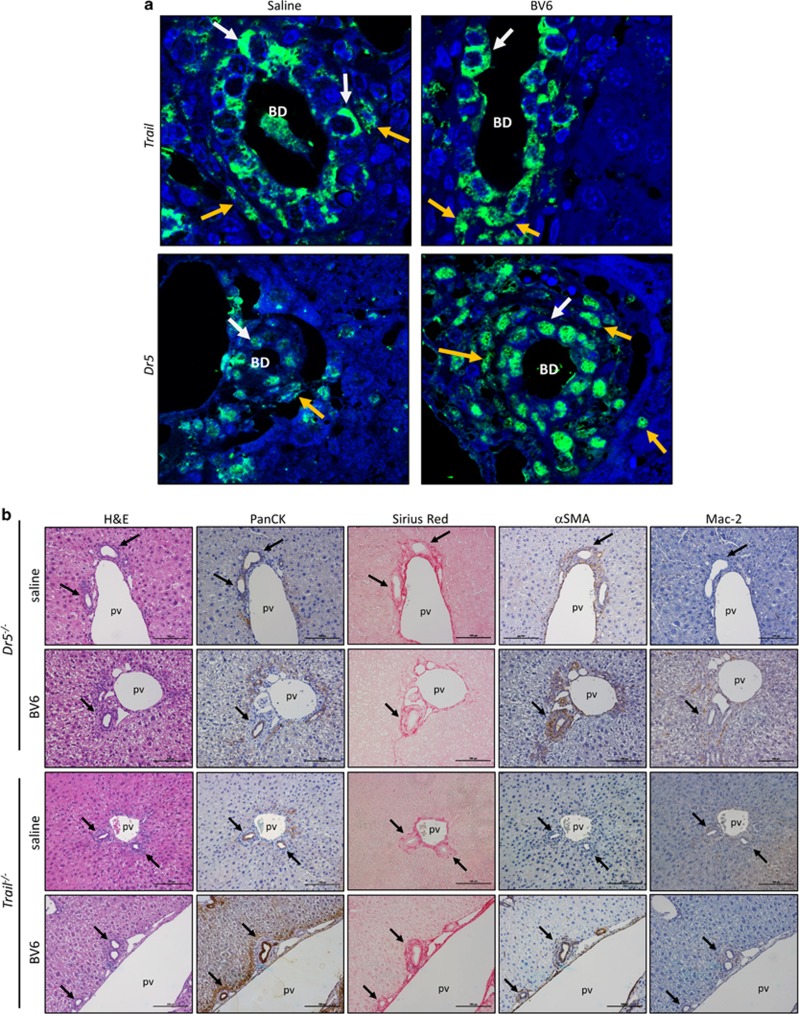

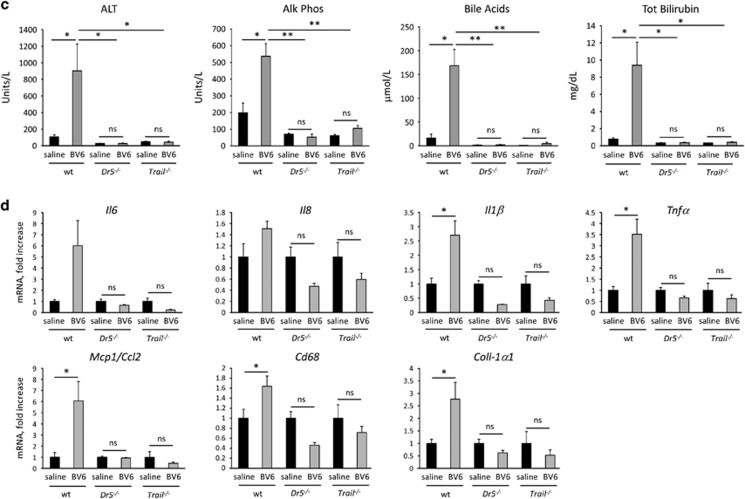
Dr5^−/−^ and Trail^−/−^ mice are resistant to biliary injury following SM intrabiliary instillation. (**a**) *In situ* hybridization using mRNA detection probes for *Trail* and *Dr5* performed on liver tissue sections of saline- or BV6-treated mice at day 5. The white arrows point to cholangiocytes. The orange arrows point to surrounding infiltrating cells (inflammatory cells, fibroblasts); BD, bile duct. (**b**) Representative images of liver sections from *Dr5*^*/-*^ (top) and *Trail*^−/−^ (bottom) mice injected with a single dose of BV6 or saline in the biliary tree and killed after 5 days. H&E staining, panCK IHC, picrosirius red-stain (collagen), *α*-smooth muscle actin IHC and Mac2 staining are depicted. Photomicrographs taken at × 20 magnification. The arrows point to the bile ducts; pv, portal vein. (**c**) Serum ALT, alkaline phosphatase, total bile acids and total bilirubin. *Dr5*^−/−^: saline (*n*=5), BV6 (*n*=5); *Trail*^−/−^: saline (*n*=4), BV6 (*n*=7). *P<0.05. (**d**) *Il6*, *Il8*, *Il1β*, *Tnfα*, *Mcp-1/Ccl2*, *Cd68 and Coll-1α1* gene expression analyzed by qPCR in total liver lysates of wild-type, *Dr5*^−/−^ and *Trail*^−/−^ mice. Expression normalized to 18S rRNA. Data are expressed as fold increase over control. Mean±S.E. are depicted. *P<0.05, **P<0.005; NS, not significant

TRAIL is expressed by a variety of immune cells, including activated macrophages, activated B and T lymphocytes, natural killer (NK) cells and neutrophils.^25, 26, 27, 28^ *In situ* hybridization analysis of mouse liver sections indicates that both TRAIL and the murine TRAIL receptor/DR5 are expressed in cholangiocytes of mice treated with saline or BV6, as well as in other infiltrating inflammatory cells and fibroblasts (Figure 7a). Given the potential role of TRAIL in cholangitis, we next explored whether TRAIL signaling is involved in the development of the SM-induced cholangiopathy. Mice deficient in DR5 (*Dr5*^−/−^) or TRAIL (*Trail*^−/−^) received biliary instillation of BV6 or saline solution as previously described. In striking contrast to our observations in the wild-type mice, *Dr5*^−/−^ and *Trail*^−/−^ mice were highly resistant to the SM-induced liver injury and displayed normal liver histology (Figure 7b). Serum markers of liver injury and cholestasis were not significantly different in *Dr5*^−/−^ or *Trail*^−/−^ BV6-treated mice compared with their respective saline-injected controls (Figure 7c). However, all serum markers were significantly lower in BV6-treated *Dr5*^−/−^ and *Trail*^−/−^ mice compared with wild-type BV6-treated mice (Figure 7c). Similarly, qPCR analysis of liver lysates demonstrated a significant increase in gene expression of markers of inflammation (IL-6, IL-8, IL-1*β*, TNF*α*), macrophage accumulation and activation (MCP-1, CD68, TNF*α*) and fibrosis (Coll-1*α*1) in the livers of BV6-treated wild-type compared to saline-injected wild-type mice, but not in the livers of BV6-injected *Dr5*^−/−^ or *Trail*^−/−^ mice (Figure 7d). Collectively, these data demonstrate that acute loss of cIAPs in biliary epithelial cells is sufficient to trigger a TRAIL-dependent periductal inflammatory response and fibrogenic cascade, resulting in a PSC-like cholestatic phenotype.

## Discussion

The current study provides mechanistic insights regarding the role of cIAPs and the TRAIL/TRAIL-R system in the development of sclerosing cholangitis. Our data indicate the following: (i) cIAP expression is significantly reduced in biliary epithelial cells of PSC patients; (ii) downregulation of cIAPs in normal human cholangiocyte cell lines by a SMAC mimetic causes moderate apoptosis and activates NF-*κ*B through the non-canonical pathway, resulting in transcriptional upregulation of several pro-inflammatory cytokines and chemokines; (iii) instillation of a SMAC mimetic into the biliary tree of C57BL/6 mice causes an acute and transient fibrous cholangiopathy; and (iv) *Dr5*^−/−^ and *Trail*^−/−^ mice are protected against the SMAC mimetic-induced cholangiopathy.

Although PSC is considered an immune-mediated disorder, its etiology and possible environmental triggers remain undefined. cIAPs are crucial regulators of cell death, inflammation and immunity; however, the extent to which cIAP-1 and cIAP-2 are involved in the pathogenesis of PSC has never been explored. Herein, we report that cIAP-1 and cIAP-2 protein levels are significantly decreased in biliary epithelial cells of patients with advanced stage PSC, as assessed by immunohistochemistry. The mechanism by which cIAPs are decreased in PSC-affected biliary epithelia is currently unclear. Signaling pathways activated by binding of TWEAK to its cognate receptor Fn14 are known to promote lysosomal degradation of cIAP-1.^19^ Our data indicate that both TWEAK and Fn14 are expressed on PSC cholangiocytes, providing a potential explanation for the loss of cIAPs.

The cIAP biology is often examined using SMAC mimetics, which bind these proteins causing their auto-ubiquitination and rapid proteasomal degradation.^13^ Downregulation of cIAPs by SMAC mimetics may lead to different outcomes. In several cancer cell lines, inhibition of IAPs results in cell death via NF-*κ*B-stimulated TNF*α* production and autocrine TNF*α*-mediated apoptosis.^12, 13^ However, loss of cIAPs may also promote apoptosis via the spontaneous formation of the ripoptosome consisting of RIP1, FADD and caspase 8, without the involvement of any death ligand and/or death receptor.^22, 29^ In addition, SMAC mimetic-mediated cIAP depletion may sensitize cells to death by other insults, particularly by the death receptor pathway.^6, 7, 30^ Our current data suggest that human cholangiocytes display limited sensitivity to SMAC mimetic-induced apoptosis, which occurs via the ripoptosome formation. Given the moderate apoptotic response, it appears unlikely that cholangiocyte cell death alone is the sole promoter of the PSC-like phenotype. Rather, early cell death with breakdown of the bile duct permeability barrier may result in bile-induced periductular inflammation. This acute inflammation may be, in part, augmented by TRAIL/TRAIL-R pro-inflammatory signals. Consistent with this interpretation of the data is the observation that a caspase inhibitor only partially attenuates the phenotype. Finally, the SMAC mimetic likely also directly affects cells besides cholangiocytes in the periductular tissue, contributing to the periductular inflammatory response.

The above observations prompted us to examine other responses that may be elicited by decreased cIAP levels. Interestingly, IAP depletion promotes generation of pro-inflammatory chemokines, such as RANTES/CCL5 and IL-8 *in vitro* and *in vivo,*^23^ likely via NF-*κ*B-mediated signaling pathways. Indeed, we observed transcriptional upregulation of pro-inflammatory cytokine and chemokine genes, such as *IL6*, *IL8*, *IL1β* and *MCP1/CCL2*, in cholangiocytes treated with a SMAC mimetic, which was largely mediated by the non-canonical activation of NF-*κ*B. These data led us to conclude that downregulation of cIAPs in cholangiocytes of PSC patients may contribute to the periductal inflammation of large bile ducts characteristic of PSC.

To verify our *in vitro* observations in an *in vivo* model, we instilled a single dose of the SMAC mimetic BV6 into the biliary system of mice. The mice displayed acute, progressive cholestatic injury resembling human sclerosing cholangitis, which peaked 5 days after surgery and completely reversed within 3 weeks. This was not unexpected, as degradation of cIAP-1 and cIAP-2 by the SMAC mimetic is transient and these proteins are rapidly restored overtime. Consistent with our findings *in vitro*, the acute injury was characterized by early onset of cholangiocyte apoptosis together with elevation of markers of inflammation and infiltration of cells of the innate immune system, such as monocytes/macrophages and neutrophils. Transcriptional upregulation of *Il8* was more significant in SM-treated cholangiocyte cell lines than in whole liver from BV6-injected mice, suggesting IL-8 may be selectively produced by cIAP-depleted cholangiocytes and may be a part of the early inflammatory response. Together with MCP-1, IL-8 is known to attract monocytes/macrophages and neutrophils to the inflammatory site.

Accumulating evidence demonstrate that, besides its apoptosis-inducing activity, TRAIL has an important role in innate immune responses and can promote inflammatory signals by inducing cytokine and chemokine expression.^31, 32^ Moreover, the TRAIL/TRAIL-receptor system has been previously implicated in the development of acute sclerosing cholangitis in mice, although the study was mostly based on data obtained following systemic injection of a TRAIL receptor agonistic antibody and did not fully examine the role of endogenous TRAIL.^5^ Therefore, it is conceivable that TRAIL may have a role in our model of SM-induced fibro-inflammatory cholangitis. In line with these findings, neither *Trail*^−/−^ mice nor *Dr5*^−/−^ mice displayed evidence of cholangiopathy after intrabiliary instillation of BV6, supporting the interpretation that ligand-dependent activation of TRAIL-receptor is a requisite for the development of the biliary injury in this model. Our observations also suggest endogenous TRAIL in the context of cIAP depletion by a SMAC mimetic can induce biliary injury. However, the specific cell type responsible for this TRAIL-mediated injury (macrophages *versus* other recruited/resident immune cells) remains to be determined. TRAIL is highly expressed in polarized macrophages, especially in the classically activated, pro-inflammatory M1 macrophages that are recruited early to the injury site.^33^ Recent studies also demonstrated that TRAIL can induce macrophage activation and polarization toward a pro-inflammatory phenotype.^31, 34^ Thus, TRAIL could potentially generate a feed-forward loop resulting in further macrophage activation, which, in turn, triggers TRAIL-mediated cytokine/chemokine generation by cholangiocytes. Finally, through their ability to directly regulate myofibroblast activation via the production of profibrotic mediators such as transforming growth factor-*β*1 (TGF-*β*1) and platelet-derived growth factor (PDGF), macrophages have been recognized as crucial regulators of the fibrotic process in the liver.^35^

Collectively, our data demonstrate that downregulation of cIAPs in cholangiocytes is sufficient to trigger cholangiocyte apoptosis and a subsequent inflammatory cascade resulting in a fibrous cholangiopathy resembling human sclerosing cholangitis. Although several questions remain to be answered to elucidate the molecular and cellular mechanisms leading to this diseased phenotype, the current study provides further insights into the role of TRAIL in the pathogenesis of sclerosing cholangitis that could be exploited for therapeutic purposes.

## Materials and Methods

### Reagents

The SMAC mimetics TL-32711 (Birinapant) and BV6 were purchased from Selleck Chemical (Houston, TX, USA). The human anti-TNF*α* neutralizing antibody (AF210) and human anti-FasL neutralizing antibody (MAB126) were from R&D Systems (Minneapolis, MN, USA). TRAIL-R2 (human):Fc (human) was from Enzo (Farmingdale, New York, NY, USA; ALX-522-005). The pan-caspase inhibitor Q-VD-OPH was from Enzyme Systems Products (Solon, OH, USA). Necrostatin-1 was purchased from Tocris Bioscience (St. Louis, MO, USA). Necrostatin 1-s was purchased from Biovision Inc. (Milpitas, CA, USA). Human recombinant TWEAK was from R&D Systems. The agonistic anti-DR5 monoclonal antibody MD5-1 was a generous gift from Dr. Hideo Yagita (Juntendo University, Tokyo, Japan). All other reagents were from Sigma-Aldrich (St. Louis, MO, USA), unless otherwise specified.

### Cell lines

The SV40-transformed normal human cholangiocyte cell line H69, low-passage, spontaneously immortalized normal human cholangiocytes (NHCs)^36^ were grown as previously described.^37^ The human breast cancer cell line MDA-MB-231 was grown in DMEM/F12 1:1 containing 10% fetal bovine serum (FBS). RIP1 knockout H69 (*RIP1*^−/−^) cells were generated using a CRISPR/Cas9 system as previously described.^31^

### Transient transfection

A small interfering RNA (siRNA) was used to silence NF*κ*B2 (p100) in NHC. Cells transfected with scramble siRNA were used as control. The cells were grown in six-well plates and transiently transfected with Lipofectamine RNAiMAX reagent (Invitrogen/Thermo Fisher Scientific, Waltham, MA, USA) using NF-*κ*B2 (ID 4791) Trilencer-27 Human siRNA (#SR303162, Origene, Rockville, MD, USA). The experiments were performed 48 h after transfection.

### Histological analysis

Twenty-eight liver tissue specimens consisting of 16 stage IV (cirrhotic stage) PSC with no evidence of cholangiocarcinoma, 12 non-alcoholic steatohepatitis (NASH) from liver explants and five normal liver tissue specimens from biopsies of patients undergoing bariatric surgery were used. The liver samples were collected with Institutional Review Board approval. Human and mouse liver tissues were fixed in 4% paraformaldehyde for 48 h, embedded in paraffin and sectioned into 4 *μ*m slices. Liver tissue sections were deparaffinized, hydrated and incubated overnight at 4 °C with the following primary antibodies: cIAP-1 (AF8181, 15 *μ*g/ml; R&D Systems); cIAP-2 (1:50; clone E40; ab32059, Abcam, Cambridge, MA, USA); TWEAK (ab199419, Abcam); Fn14 (ab109365, Abcam); PanCK (1:1000; Z0622, Dako, Carpinteria, CA, USA); *α*-smooth muscle actin (*α*SMA; 1:500, ab32575, Abcam); Mac2 (galectin-3; 1:250, eBioscience, San Diego, CA, USA); mouse neutrophil differentiation antigen (1:200; CL8993AP, Cedarlane, Burlington, NC, USA); CD3 (1:100, A0452, Dako). Bound antibodies were detected with either biotin conjugated (Vector Laboratories, Burlingame, CA, USA) or HRP conjugated secondary antibodies (Dako) using diaminobenzidine tetrahydrochoride as chromogen. Tissue slices were counterstained with hematoxylin. Hematoxylin/eosin stain was performed by standard methods. Liver fibrosis was examined using Sirius red staining as previously described.^38^ Images were obtained using a Nikon Eclipse TE300 microscope (Nikon, Tokyo, Japan) equipped with Nikon Digital Sight DS-Ri1. Quantification of immunostaining for cIAP-1 and cIAP-2 was based on numerical scores of 0, 1, 2 and 3 for absent, weak, moderate and strong staining, respectively. A representative panel for scoring is provided in Supplementary Figure 1.

### Immunoblot analysis

Whole-cell lysates were obtained and analyzed by immunoblot as previously described.^39^ The following primary antibodies were used: cIAP-1 (1:2000) from R&D Systems; anti-cIAP-2 (1:1000) and anti-IL-6 (ab6672; 1:1000) from Abcam; actin (sc-1615; 1:1000), I*κ*B*α* (sc-371; 1:1000), NIK (sc-7211; 1:1000) from Santa Cruz Biotechnology (Santa Cruz, CA, USA); RIP1 (BD 610458; 1:1000) from BD Biosciences (San Jose, CA, USA); NF-*κ*B2 p100/p52 (#4882; 1:1000) and PARP (#9532; 1:1000) from Cell Signaling Technology (Beverly, MA, USA); GAPDH (MAB374; 1:5000) from EMD Millipore (Temecula, CA, USA).

### Immunoprecipitation assay

After removing the media, the cells were washed in ice-cold PBS and solubilized in lysis buffer (50 mM HEPES (pH 7.2), 120 mM NaCl, 1 mM EDTA, 0.1% NP-40, 10% (w/v) glycerol, protease inhibitor cocktail) for 30 min on ice. The cells were then centrifuged at 13 000 × *g* for 15 min, supernatants were recovered and protein concentration was determined using the Bradford reagent (Sigma-Aldrich). Aliquots containing 4 mg of protein were incubated with 15 *μ*g of goat polyclonal anti-caspase 8 (sc-6136, Santa Cruz) antisera for 2 h at 4 °C, then incubated overnight with protein G agarose beads (rec. protein G sepharose 4B conjugate; #101241, Thermo Fisher Scientific) at 4 °C under rotary agitation. The pelleted proteins were solubilized in SDS sample buffer, boiled for 5 min, clarified by centrifugation and subjected to SDS-PAGE and immunoblot analysis using the following primary antibodies: caspase 8 (#9746; 1:1000, Cell Signaling Technology), RIP1, FADD (sc-6035, 1:500, Santa Cruz Biotechnology).

### Apoptosis assays

Apoptosis was assessed morphologically by fluorescence microscopy (Nikon Eclipse TE200, Nikon Instruments Inc., Tokyo, Japan) after staining with 4′,6-diamidino-2-phenylindole dihydrochloride (DAPI, Sigma) as previously described^40^ and biochemically by measuring caspase-3/7 activity in cell cultures using the Apo-ONE homogeneous caspase-3/7 kit (Promega, Madison, WI, USA) following the supplier's instructions.

### Animal studies

All animal experiments were performed in accordance with protocols approved by the Mayo Clinic Institutional Animal Care and Use Committee. C57BL/6J mice were obtained from Jackson Labs (Bar Harbor, ME, USA). TRAIL receptor knockout mice (*Dr5*^−/−^ on a C57Bl/6J background) were a generous gift from Dr. Wafik S. El-Deiry (Pennsylvania State University, PA, USA).^41^ TRAIL knockout mice (*Trail*^−/−^) were generated as previously described.^42^ Wild-type mice were not housed together with either *Dr5*^−/−^ or *Trail*^−/−^ mice. Six-to-eight-week-old male mice were anesthetized by intraperitoneal (i.p.) pentobarbital injections (40–85 mg/kg). Under deep anesthesia, the abdominal cavity was opened by a midline approach and the liver gently retracted and allowed to rest on the diaphragm. The common bile duct (CBD) located below the liver was clamped with a small animal surgical clip (00396-01; F.S.T., Foster City, CA, USA) to prevent the injected material from rapidly flowing into the duodenum. One hundred *μ*l of BV6 solution (0.1 mg/100 *μ*l in saline solution) or saline solution (control) were injected into the gallbladder with enough pressure to allow the solution to distend the biliary system. On withdrawal of the needle, a sterile cotton-tipped applicator was held over the injection site for about 1 min to prevent leakage. The mice were kept under anesthesia for 45 min, then the CBD was unclamped and the internal organs were returned to their original position. The abdominal wall and skin were closed in separate layers with absorbable chromic 3-0 gut suture material. At day 5, the mice were anesthetized using a combination of xylazine (120 mg/kg, i.p.) and ketamine (10 mg/kg, i.p.), blood was collected from the inferior vena cava and the liver harvested for histological analysis. In a subset of animals, the liver was additionally perfused with heparinized phosphate buffered saline and used for generating cholangiograms as described below.

### Cholangiograms

Briefly, the CBD was exposed and catheterized with 0.15 mm ID × 0.3 mm OD polytetrafluoroethylene tubing (Braintree Scientifics Inc., Braintree, MA, USA). A lead chromate containing radio-opaque silicone polymer, Microfils (MV122, Flowtech, Carver, MA, USA), composed of a mixture of MV compound, MV diluent and MV curing agent was injected into the bile ducts retrogradely through the CBD at constant pressure. When the lobes were filled, the CBD was ligated and the polymer was allowed to set *in situ* at room temperature for 20 min. The gall bladder was resected to ensure better visualization of the biliary tree. The liver was then dissected out and transferred to 4% buffered formalin. Complete setting was achieved overnight at 4 °C. The formalin-preserved liver was immobilized by embedding in paraffin wax and imaged in a micro-CT scanner over a period of 11 h using a molybdenum X-ray anode and zirconium X-ray filter, at a pixel size setting of 19.7 *μ*m at axis of rotation. Imaging was carried out at the Physiological Imaging Research Lab (Mayo Clinic Rochester, MN, USA). The X-ray projection data comprising 721 slices over a 360-degree rotation was subjected to standard filtered-back projection tomographic reconstruction to generate the three-dimensional image of the intrahepatic biliary tree.

### Serum analysis

Serum alanine aminotransferase (ALT), alkaline phosphatase, total bile acids and total bilirubin were measured using a commercially available veterinary chemistry analyzer (VetScan 2, Abaxis, Union City, CA, USA).

### Reverse transcription-quantitative polymerase chain reaction (RT-qPCR)

Total RNA was isolated from cell cultures and frozen liver tissue using RNeasy Plus mini kit (Qiagen, Hilden, Germany). Reverse transcription was performed using Moloney murine leukemia virus reverse transcriptase and random primers (both Invitrogen, Carlsbad, CA, USA). Real-time PCR for quantification of the cDNA template was performed on Light Cycler 480 (Roche, Indianapolis, IN, USA) using SYBR green (Roche) as the fluorophore. Target gene expression was calculated using ΔΔCt method and expression was normalized to 18S rRNA or GAPDH RNA.^43^ The primers used are listed in Supplementary Tables 1 and 2.

### Terminal deoxynucleotidyl transferase dUTP nick end-labeling assay

The TUNEL assay was performed on paraffin-embedded tissue sections using the commercially available ApopTag Peroxidase *in situ* Apoptosis Detection Kit (EMD Millipore) following the manufacturer's protocol. The images were obtained using a Zeiss AX10 microscope equipped with an Axiocam 105 color camera (Carl Zeiss, Oberkochen, Germany).

### Transmission electron microscopy

The mouse liver samples were fixed in 1% osmium tetroxide for 1 h, rinsed in distilled water, dehydrated, embedded in Spurr's resin and sectioned at 80 nm. The images were taken using a JEOL 1200 electron microscope (JEOL USA, Peabody, MA, USA).

### Fluorescence *in situ* hybridization

Localization of *Dr5* and *Trail* mRNA in mouse liver was determined using FISH on 4-6 *μ*m tissue sections cut from paraffin embedded liver followed by tyramide amplification. Custom locked nucleic acid (LNA) mRNA detection probes were designed by and purchased from Exiqon (Woburn, MA, USA). The protocol was adapted from previously described methods.^44^ Briefly, the liver tissue sections were deparaffinized and hydrated with antigen retrieval performed in boiling citrate buffer (pH 6.0). Probes were hybridized at 60 °C for 1 h. For tyramide amplification, tissue sections were blocked, endogenous peroxidase was quenched and tissues were incubated overnight at 4 °C with anti-fluorescein peroxidase-conjugated antibody (Rockland Immunochemicals, Gilbertsville, PA, USA) at a dilution of 1:1000. The sections were incubated in tyramide amplification solution (Perkin Elmer, Waltham, MA, USA) and subsequently cover-slipped using Prolong Gold with DAPI (Invitrogen/Thermo Fisher Scientific). Negative controls with hybridization buffer alone were carried out in parallel. The images were taken on a confocal microscope Zeiss LSM 510 confocal microscope (Carl Zeiss) with a × 63 water objective.

### Statistical analysis

All the data are indicated as mean (or fold change in mean over control)±standard error (S.E.). Statistical analyses were performed with two-tailed Student's *t*-test and *P*<0.05 were considered statistically significant.

## References

[bib1] Eaton JE, Talwalkar JA, Lazaridis KN, Gores GJ, Lindor KD. Pathogenesis of primary sclerosing cholangitis and advances in diagnosis and management. Gastroenterology 2013; 145: 521–536. 23827861 10.1053/j.gastro.2013.06.052 PMC3815445

[bib2] Alabraba E, Nightingale P, Gunson B, Hubscher S, Olliff S, Mirza D et al. A re-evaluation of the risk factors for the recurrence of primary sclerosing cholangitis in liver allografts. Liver Transpl 2009; 15: 330–340. 19243003 10.1002/lt.21679

[bib3] Fickert P, Zollner G, Fuchsbichler A, Stumptner C, Weiglein AH, Lammert F et al. Ursodeoxycholic acid aggravates bile infarcts in bile duct-ligated and Mdr2 knockout mice via disruption of cholangioles. Gastroenterology 2002; 123: 1238–1251. 12360485 10.1053/gast.2002.35948

[bib4] Pollheimer MJ, Trauner M, Fickert P. Will we ever model PSC? – ‘it's hard to be a PSC model!'. Clin Res Hepatol Gastroenterol 2011; 35: 792–804. 21703962 10.1016/j.clinre.2011.04.014

[bib5] Takeda K, Kojima Y, Ikejima K, Harada K, Yamashina S, Okumura K et al. Death receptor 5 mediated-apoptosis contributes to cholestatic liver disease. Proc Natl Acad Sci USA 2008; 105: 10895–10900. 18667695 10.1073/pnas.0802702105 PMC2504811

[bib6] Li L, Thomas RM, Suzuki H, De Brabander JK, Wang X, Harran PG. A small molecule Smac mimic potentiates TRAIL- and TNFalpha-mediated cell death. Science 2004; 305: 1471–1474. 15353805 10.1126/science.1098231

[bib7] Fulda S. Molecular pathways: targeting death receptors and smac mimetics. Clin Cancer Res 2014; 20: 3915–3920. 24824309 10.1158/1078-0432.CCR-13-2376

[bib8] Varfolomeev E, Goncharov T, Fedorova AV, Dynek JN, Zobel K, Deshayes K et al. c-IAP1 and c-IAP2 are critical mediators of tumor necrosis factor alpha (TNFalpha)-induced NF-kappaB activation. J Biol Chem 2008; 283: 24295–24299. 18621737 10.1074/jbc.C800128200 PMC3259840

[bib9] Varfolomeev E, Goncharov T, Maecker H, Zobel K, Komuves LG, Deshayes K et al. Cellular inhibitors of apoptosis are global regulators of NF-kappaB and MAPK activation by members of the TNF family of receptors. Sci Signal 2012; 5: ra22. 22434933 10.1126/scisignal.2001878

[bib10] Hayden MS, Ghosh S. Shared principles in NF-kappaB signaling. Cell 2008; 132: 344–362. 18267068 10.1016/j.cell.2008.01.020

[bib11] Perkins ND. Integrating cell-signalling pathways with NF-kappaB and IKK function. Nat Rev Mol Cell Biol 2007; 8: 49–62. 17183360 10.1038/nrm2083

[bib12] Vince JE, Wong WW, Khan N, Feltham R, Chau D, Ahmed AU et al. IAP antagonists target cIAP1 to induce TNFalpha-dependent apoptosis. Cell 2007; 131: 682–693. 18022363 10.1016/j.cell.2007.10.037

[bib13] Varfolomeev E, Blankenship JW, Wayson SM, Fedorova AV, Kayagaki N, Garg P et al. IAP antagonists induce autoubiquitination of c-IAPs, NF-kappaB activation, and TNFalpha-dependent apoptosis. Cell 2007; 131: 669–681. 18022362 10.1016/j.cell.2007.10.030

[bib14] Vallabhapurapu S, Matsuzawa A, Zhang W, Tseng PH, Keats JJ, Wang H et al. Nonredundant and complementary functions of TRAF2 and TRAF3 in a ubiquitination cascade that activates NIK-dependent alternative NF-kappaB signaling. Nat Immunol 2008; 9: 1364–1370. 18997792 10.1038/ni.1678 PMC2671996

[bib15] Varfolomeev E, Vucic D. (Un)expected roles of c-IAPs in apoptotic and NFkappaB signaling pathways. Cell Cycle 2008; 7: 1511–1521. 18469528 10.4161/cc.7.11.5959

[bib16] Xiao G, Harhaj EW, Sun SC. NF-kappaB-inducing kinase regulates the processing of NF-kappaB2 p100. Mol Cell 2001; 7: 401–409. 11239468 10.1016/s1097-2765(01)00187-3

[bib17] Tang W, Wang W, Zhang Y, Liu S, Liu Y, Zheng D. TRAIL receptor mediates inflammatory cytokine release in an NF-kappaB-dependent manner. Cell Res 2009; 19: 758–767. 19434100 10.1038/cr.2009.57

[bib18] Vince JE, Silke J. TWEAK shall inherit the earth. Cell Death Differ 2006; 13: 1842–1844. 16932755 10.1038/sj.cdd.4402027

[bib19] Vince JE, Chau D, Callus B, Wong WW, Hawkins CJ, Schneider P et al. TWEAK-FN14 signaling induces lysosomal degradation of a cIAP1-TRAF2 complex to sensitize tumor cells to TNFalpha. J Cell Biol 2008; 182: 171–184. 18606850 10.1083/jcb.200801010 PMC2447903

[bib20] Brumatti G, Ma C, Lalaoui N, Nguyen NY, Navarro M, Tanzer MC et al. The caspase-8 inhibitor emricasan combines with the SMAC mimetic birinapant to induce necroptosis and treat acute myeloid leukemia. Sci Transl Med 2016; 8: 339ra369. 10.1126/scitranslmed.aad3099 27194727

[bib21] Condon SM, Mitsuuchi Y, Deng Y, LaPorte MG, Rippin SR, Haimowitz T et al. Birinapant, a smac-mimetic with improved tolerability for the treatment of solid tumors and hematological malignancies. J Med Chem 2014; 57: 3666–3677. 24684347 10.1021/jm500176w

[bib22] Tenev T, Bianchi K, Darding M, Broemer M, Langlais C, Wallberg F et al. The Ripoptosome, a signaling platform that assembles in response to genotoxic stress and loss of IAPs. Mol Cell 2011; 43: 432–448. 21737329 10.1016/j.molcel.2011.06.006

[bib23] Kearney CJ, Sheridan C, Cullen SP, Tynan GA, Logue SE, Afonina IS et al. Inhibitor of apoptosis proteins (IAPs) and their antagonists regulate spontaneous and tumor necrosis factor (TNF)-induced proinflammatory cytokine and chemokine production. J Biol Chem 2013; 288: 4878–4890. 23275336 10.1074/jbc.M112.422410 PMC3576092

[bib24] Darding M, Feltham R, Tenev T, Bianchi K, Benetatos C, Silke J et al. Molecular determinants of Smac mimetic induced degradation of cIAP1 and cIAP2. Cell Death Differ 2011; 18: 1376–1386. 21331077 10.1038/cdd.2011.10 PMC3172091

[bib25] Griffith TS, Wiley SR, Kubin MZ, Sedger LM, Maliszewski CR, Fanger NA. Monocyte-mediated tumoricidal activity via the tumor necrosis factor-related cytokine, TRAIL. J Exp Med 1999; 189: 1343–1354. 10209050 10.1084/jem.189.8.1343 PMC2193036

[bib26] Simons MP, Leidal KG, Nauseef WM, Griffith TS. TNF-related apoptosis-inducing ligand (TRAIL) is expressed throughout myeloid development, resulting in a broad distribution among neutrophil granules. J Leukoc Biol 2008; 83: 621–629. 18063697 10.1189/jlb.0707452

[bib27] Mariani SM, Krammer PH. Surface expression of TRAIL/Apo-2 ligand in activated mouse T and B cells. Eur J Immunol 1998; 28: 1492–1498. 9603453 10.1002/(SICI)1521-4141(199805)28:05<1492::AID-IMMU1492>3.0.CO;2-X

[bib28] Falschlehner C, Schaefer U, Walczak H. Following TRAIL's path in the immune system. Immunology 2009; 127: 145–154. 19476510 10.1111/j.1365-2567.2009.03058.x PMC2691779

[bib29] Feoktistova M, Geserick P, Kellert B, Dimitrova DP, Langlais C, Hupe M et al. cIAPs block Ripoptosome formation, a RIP1/caspase-8 containing intracellular cell death complex differentially regulated by cFLIP isoforms. Mol Cell 2011; 43: 449–463. 21737330 10.1016/j.molcel.2011.06.011 PMC3163271

[bib30] Griffith TS, Kucaba TA, O'Donnell MA, Burns J, Benetatos C, McKinlay MA et al. Sensitization of human bladder tumor cells to TNF-related apoptosis-inducing ligand (TRAIL)-induced apoptosis with a small molecule IAP antagonist. Apoptosis 2011; 16: 13–26. 20734142 10.1007/s10495-010-0535-3

[bib31] Hirsova P, Ibrahim SH, Krishnan A, Verma VK, Bronk SF, Werneburg NW et al. Lipid-induced signaling causes release of inflammatory extracellular vesicles from hepatocytes. Gastroenterology 2016; 150: 956–967. 26764184 10.1053/j.gastro.2015.12.037 PMC4808464

[bib32] Cullen SP, Martin SJ. Fas and TRAIL 'death receptors' as initiators of inflammation: Implications for cancer. Semin Cell Dev Biol 2015; 39: 26–34. 25655947 10.1016/j.semcdb.2015.01.012

[bib33] Martinez FO, Gordon S, Locati M, Mantovani A. Transcriptional profiling of the human monocyte-to-macrophage differentiation and polarization: new molecules and patterns of gene expression. J Immunol 2006; 177: 7303–7311. 17082649 10.4049/jimmunol.177.10.7303

[bib34] Gao J, Wang D, Liu D, Liu M, Ge Y, Jiang M et al. Tumor necrosis factor-related apoptosis-inducing ligand induces the expression of proinflammatory cytokines in macrophages and re-educates tumor-associated macrophages to an antitumor phenotype. Mol Biol Cell 2015; 26: 3178–3189. 26224317 10.1091/mbc.E15-04-0209 PMC4569310

[bib35] Wynn TA, Barron L. Macrophages: master regulators of inflammation and fibrosis. Semin Liver Dis 2010; 30: 245–257. 20665377 10.1055/s-0030-1255354 PMC2924662

[bib36] Banales JM, Saez E, Uriz M, Sarvide S, Urribarri AD, Splinter P et al. Up-regulation of microRNA 506 leads to decreased Cl-/HCO3- anion exchanger 2 expression in biliary epithelium of patients with primary biliary cirrhosis. Hepatology 2012; 56: 687–697. 22383162 10.1002/hep.25691 PMC3406248

[bib37] Grubman SA, Perrone RD, Lee DW, Murray SL, Rogers LC, Wolkoff LI et al. Regulation of intracellular pH by immortalized human intrahepatic biliary epithelial cell lines. Am J Physiol 1994; 266: G1060–G1070. 8023938 10.1152/ajpgi.1994.266.6.G1060

[bib38] Krishnan A, Li X, Kao WY, Viker K, Butters K, Masuoka H et al. Lumican, an extracellular matrix proteoglycan, is a novel requisite for hepatic fibrosis. Lab Invest 2012; 92: 1712–1725. 23007134 10.1038/labinvest.2012.121 PMC3810270

[bib39] Guicciardi ME, Bronk SF, Werneburg NW, Yin XM, Gores GJ. Bid is upstream of lysosome-mediated caspase 2 activation in tumor necrosis factor alpha-induced hepatocyte apoptosis. Gastroenterology 2005; 129: 269–284. 16012953 10.1053/j.gastro.2005.05.022

[bib40] Guicciardi ME, Mott JL, Bronk SF, Kurita S, Fingas CD, Gores GJ. Cellular inhibitor of apoptosis 1 (cIAP-1) degradation by caspase 8 during TNF-related apoptosis-inducing ligand (TRAIL)-induced apoptosis. Exp Cell Res 2011; 317: 107–116. 20951133 10.1016/j.yexcr.2010.10.005 PMC2991414

[bib41] Wu GS, Burns TF, Zhan Y, Alnemri ES, El-Deiry WS. Molecular cloning and functional analysis of the mouse homologue of the KILLER/DR5 tumor necrosis factor-related apoptosis-inducing ligand (TRAIL) death receptor. Cancer Res 1999; 59: 2770–2775. 10383128

[bib42] Cretney E, Takeda K, Yagita H, Glaccum M, Peschon JJ, Smyth MJ. Increased susceptibility to tumor initiation and metastasis in TNF-related apoptosis-inducing ligand-deficient mice. J Immunol 2002; 168: 1356–1361. 11801676 10.4049/jimmunol.168.3.1356

[bib43] Lutfalla G, Uze G. Performing quantitative reverse-transcribed polymerase chain reaction experiments. Methods Enzymol 2006; 410: 386–400. 16938562 10.1016/S0076-6879(06)10019-1

[bib44] de Planell-Saguer M, Rodicio MC, Mourelatos Z. Rapid *in situ* codetection of noncoding RNAs and proteins in cells and formalin-fixed paraffin-embedded tissue sections without protease treatment. Nat Protoc 2010; 5: 1061–1073. 20539282 10.1038/nprot.2010.62

