# Supplementary material for: Biliary tract instillation of a SMAC mimetic induces TRAIL-dependent acute sclerosing cholangitis-like injury in mice
Source: Cell Death Dis. 2017 Jan 5;8(1):e2535–. doi: 10.1038/cddis.2016.459 (PMC5386369; doi:10.1038/cddis.2016.459)
Supplement: Supplementary Figure Legends [file cddis2016459x3.pdf]

SUPPLEMENTARY FIGURE LEGENDS

Figure S1. Example of histological scoring system used to grade cIAP-1 and cIAP-2 immunostaining in human liver sections.

Figure S2. **(A)** Apoptosis assessed by DAPI staining (left panels) and by caspase 3/7 activation (right panels) in human cholangiocyte cell lines H69 and NHC, and the human breast cancer cell line MDA-MB-231, incubated for 24 hr with or without (cnt) the SMAC mimetic BV6 (5 μM), in the presence or absence of neutralizing antibodies against TNFα (1μg/ml) or FasL (1μg/ml), or recombinant TRAIL-R2:Fc (1μg/ml). **(B)** *TNFα* and *TRAIL* gene expression analyzed by qPCR in H69, NHC and MDA-MB-231 cells incubated in the presence of BV6 for the indicated times. Expression normalized to GAPDH RNA. Data are expressed as fold increase over control. Mean ± s.e. are depicted from 3 independent experiments in H69 and NHC.
