# Supplementary material for: Biliary tract instillation of a SMAC mimetic induces TRAIL-dependent acute sclerosing cholangitis-like injury in mice
Source: Cell Death Dis. 2017 Jan 5;8(1):e2535–. doi: 10.1038/cddis.2016.459 (PMC5386369; doi:10.1038/cddis.2016.459)
Supplement: Supplementary Table 1 and 2 [file cddis2016459x4.pdf]

Supplementary Table 1. qPCR Human Primers.

**Gene-specific Primer**	**Sequences (5' → 3')**
IL-1β - forward	ATGATGGCTTATTACAGTGGCAA
IL-1β - reverse	GTCGGAGATTCGTAGCTGGA
IL-6 - forward	ACTCACCTCTTCAGAACGAATTG
IL-6 - reverse	CCATCTTTGGGAGGTTCAGGTTG
IL-8 - forward	GGCACAAACTTTCAGAGACAG
IL-8 - reverse	ACACAGAGCTGCAGAAATCAGG
MCP-1 - forward	CAGCCAGATGCAATCAATGCC
MCP-1 - reverse	TGGAATCCTGAACCCACTTCT
RANTES - forward	TTTGCCTACATTGCCCGC
RANTES - reverse	TTTCGGGTGACAAAGACGACT
TNFα - forward	CAGAGGGCCTGTACCTCATC
TNFα - reverse	GGAAGACCCCTCCCAGATAG
TRAIL - forward	TGCGTGCTGATCGTGATCTTC
TRAIL - reverse	GCTCGTTGGTAAAGTACACGTA
FasL - forward	TCTGGAATGGGAAGACACC
FasL - reverse	CACATCTGCCCAGTAGTGC
GAPDH - forward	CCAGGGCTGCTTTTAACTCT
GAPDH - reverse	GGACTCCACGACGTACTCA
18s - forward	CGCTTCCTTACCTGGTTGAT
18s - reverse	GAGCGACCAAAGGAACCATA

Supplementary Table 2. qPCR Mouse Primers.

**Gene-specific Primer**	**Sequences (5' → 3')**
IL-1β - forward	GCAACTGTTCCTGAACTCAACT
IL-1β - reverse	ATCTTTTGGGGTCCGTCAACT
IL-6 - forward	TAGTCCTTCCTACCCCAATTTCC
IL-6 - reverse	TTGGTCCTTAGCCACTCCTTC
IL-8 - forward	CCTACTTCAGCATCCTCTACTGG
IL-8 - reverse	AGGGTTTCTTGAGAAGGGGAC
CD68 - forward	TGTCTGATCTTGCTAGGACCG
CD68 - reverse	GAGAGTAACGGCCTTTTTGTGA
MCP-1 - forward	TTAAAAACCTGGATCGGAACCAA
MCP-1 - reverse	GCATTAGCTTCAGATTTACGGGT
Coll1A1 - forward	GCTCCTCTTAGGGGCCACT
Coll1A1 - reverse	CCACGTCTCACCATTGGGG
TNFα - forward	CCCTCACACTCAGATCATCTTCT
TNFα - reverse	GCTACGACGTGGGCTACAG
18s - forward	CGCTTCCTTACCTGGTTGAT
18s - reverse	GAGCGACCAAAGGAACCATA
